# Cross-Streams Through the Ventral Posteromedial Thalamic Nucleus to Convey Vibrissal Information

**DOI:** 10.3389/fnana.2021.724861

**Published:** 2021-10-28

**Authors:** Huimin Zhang, Xiaojun Wang, Wenyan Guo, Anan Li, Ruixi Chen, Fei Huang, Xiaoxiang Liu, Yijun Chen, Ning Li, Xiuli Liu, Tonghui Xu, Zheng Xue, Shaoqun Zeng

**Affiliations:** ^1^Collaborative Innovation Center for Biomedical Engineering, Wuhan National Laboratory for Optoelectronics-Huazhong University of Science and Technology, Wuhan, China; ^2^Britton Chance Center and MOE Key Laboratory for Biomedical Photonics, School of Engineering Sciences, Huazhong University of Science and Technology, Wuhan, China; ^3^Department of Laboratory Animal Science, Fudan University, Shanghai, China; ^4^Department of Neurology, Tongji Hospital, Tongji Medical College, Huazhong University of Science and Technology, Wuhan, China

**Keywords:** barrel columns, ventral posteromedial thalamic nucleus, complete neuron morphology, trigeminal nucleus interpolaris, parallel pathways, cross-streams

## Abstract

Whisker detection is crucial to adapt to the environment for some animals, but how the nervous system processes and integrates whisker information is still an open question. It is well-known that two main parallel pathways through Ventral posteromedial thalamic nucleus (VPM) ascend to the barrel cortex, and classical theory suggests that the cross-talk from trigeminal nucleus interpolaris (Sp5i) to principal nucleus (Pr5) between the main parallel pathways contributes to the multi-whisker integration in barrel columns. Moreover, some studies suggest there are other cross-streams between the parallel pathways. To confirm their existence, in this study we used a dual-viral labeling strategy and high-resolution, large-volume light imaging to get the complete morphology of individual VPM neurons and trace their projections. We found some new thalamocortical projections from the ventral lateral part of VPM (VPMvl) to barrel columns. In addition, the retrograde-viral labeling and imaging results showed there were the large trigeminothalamic projections from Sp5i to the dorsomedial section of VPM (VPMdm). Our results reveal new cross-streams between the parallel pathways through VPM, which may involve the execution of multi-whisker integration in barrel columns.

## Introduction

How the brain builds up a representation of the surrounding world is crucial in neuroscience (Diamond et al., 2008), and the study of sensory systems helps reveal the mechanism by which the nervous system processes and responds to specific sensory input (Feldmeyer et al., 2013). So far, the rodent whisker sensory system with its modular has become an ideal model to study perceptual mechanism, and it has achieved results on neural properties and synaptic connectivity of whisker sensory system (Fox, 2008; Kanold and Luhmann, 2010; Feldmeyer et al., 2013).

The whisker arrangement and the corresponding barrel columns are one of the best paradigms for studying sensory integration (Diamond et al., 2008). The multi-whisker integration is achieved from multi-whisker receptive fields, including the surround receptive fields of the cells with adjacent whisker response and/or receptive field of the cells with a single whisker response (Simons, 1985; Moore and Nelson, 1998; Zhu and Connors, 1999; Petersen and Diamond, 2000; Fox et al., 2003; Kwegyir-Afful et al., 2005). Previous studies demonstrated that sparse intracortical transmission connections in the cortex plays minorly a role in multi-whisker receptive fields in barrel columns (Simons and Woolsey, 1979; Kwegyir-Afful et al., 2005). Besides, the subcortical pathways determine primarily this multi-whisker integration of barrel columns (Timofeeva et al., 2004; Kwegyir-Afful et al., 2005). Specifically, the inputs of ventral posterior medial nucleus (VPM) neurons are involved in multi-whisker integration of barrel columns directly (Kwegyir-Afful et al., 2005). There are two mainly typically parallel streams through VPM (Pierret et al., 2000). One pathway is that the mono-whisker lemniscal (lemniscal1) having principal whisker responses (PW) arises from Pr5, transmits to the core of the VPM region (VPMc), and then terminates in the barrel columns (Lu and Lin, 1993; Agmon et al., 1995; Veinante and Deschênes, 1999; Pierret et al., 2000). The other pathway is that the extralemniscal having multi-whisker property (AW) originates from multi-whisker Sp5i, transfers to the multi-whisker ventral lateral part of the VPM (VPMvl), and then ascends to septa and S2 in the cortex (Kwegyir-Afful et al., 2005). Each pathway of parallel streams alone is not equal to constitute the subcortical pathway through VPM to implement multi-whisker integration of barrel columns. Interestingly, there is a cross-talk that happens from Sp5i to principal nucleus Pr5 in the brainstem between the parallel streams (Timofeeva et al., 2004; Kwegyir-Afful et al., 2005). This cross-talk endues typical parallel streams through VPM, the structural basis to multi-whisker integration of barrel columns (Kwegyir-Afful et al., 2005). Its existence explains the circuit mechanism for subcortical multi-whisker integration in barrel columns. The current research shows that the circuit foundation for multi-whisker integration is very complicated and needs to be further revealed.

Although some studies reported other cross-streams between the two main typical parallel pathways (Saporta and Kruger, 1977; Pierret et al., 2000; Timofeeva et al., 2004), such as the ascending projections from the VPMvl region to the single barrel column (Saporta and Kruger, 1977), the cross-streams still were controversy. On the other side, ultrastructural and electrophysiological studies showed that VPM neurons accept projections from both Pr5 and Sp5i simultaneously, but the precise location of these neurons in the VPM remains undetermined (Wang and Ohara, 1993; Friedberg et al., 1999; Pierret et al., 2000). As we know that the Pr5 neurons innervate the dorsomedial section of VPM (VPMdm), including VPMc and the head of VPM region (VPMh) (Pierret et al., 2000; Veinante et al., 2000a,b). Some Sp5i neurons have terminals that form a shell at the margin of the VPM next to Pom, but have no axons found to innervate this part of the VPM region based on incompletely individual Sp5i neuron reconstructions (Williams et al., 1994; Veinante et al., 2000a; Urbain and Deschênes, 2007). In short, there might be cross-streams between typically parallel pathways at VPM, namely the outputs of VPMvl projecting to barrel columns and the inputs of VPMc received from Sp5i.

In this study, a sparsely and brightly dual-virus strategy including an anterogradely Cre-dependent adenoassociated virus (AAV) was used to label individual VPM neurons, and their whole morphologies were acquired by the high-resolution chemical sectioning fluorescence tomography (CSFT) system (Wang et al., 2020). Results showed some VPMvl neurons sent main projections to barrel columns. And further, VPM neurons were classified into three types basing on their soma position and thalamocortical projection patterns. An optimized retrograde-viral strategy including a Cre-dependent adenoassociated virus (AAV) was employed to trace Sp5i projection, and results showed VPMc neurons received inputs from Sp5i neurons. Therefore, we demonstrated two new crosses between the two mainly parallelly thalamic pathways in VPM. These cross-streams could mediate the multi-whisker receptive fields of barrel columns in the whisker sensory system. These cross-streams could relay the multi-whisker information through VPM nuclei to barrel columns for mice to navigate and explore surrounding complex information.

## Materials and Methods

### Stereotaxic Virus Injection

**Figure 1:**
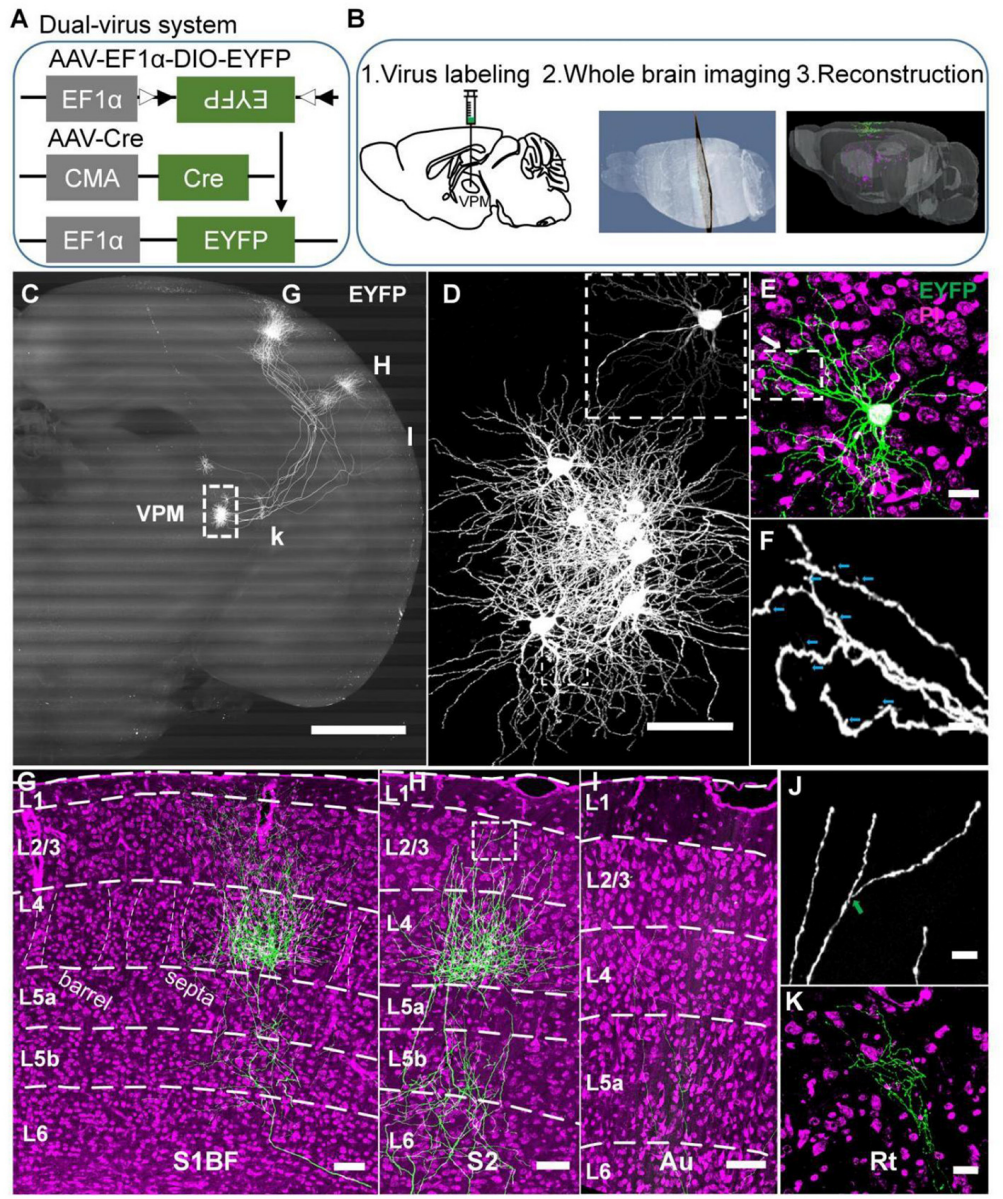
A strategy for achieving complete morphologies of individual VPM neurons. **(A)** Schematic diagram of the dual-virus system. **(B)** Procedures to obtain individual VPM neurons. Step 1, virus injection in VPM; step 2, slicing and imaging brain with CSFT system; step 3, neuron reconstruction. **(C)** The overall projection of VPM neurons in the whole brain. The axons project to RT, S1BF, S2, and Au. 800-μm max-intensity projection. **(D)** The VPM cell bodies. The amplification from the white dashed box in **(C)**. **(E)** Morphology of VPM dendrites. Amplification of EYFP channel image from the white dashed box in **(D)**. Five-μm max-intensity projection. Green: 300-μm max-intensity projection. **(F)** Distal dendrites of VPM neuron including closely dendritic excrescences and spines. The amplification from the white box in **(E)**. Cyan arrows represent spines. **(G)** Laminar distribution of VPM axons in S1BF. White dashed curves in layer 4: the borders between barrels and septa. **(H)** Laminar distribution of VPM axons in S2. **(I)** Laminar distribution of VPM axons in Au. **(J)** Remote VPM axons. Amplification of EYFP channel image from the white box in **(H)**. Green arrow: branch point. **(K)** VPM axons in Rt. Scale bar: 1,000 μm **(C)**; 100 μm **(D)**; 100 μm **(E)**; 25 μm **(F)**; 200 μm **(G–I)**; 20 μm **(J)**; 50 μm **(K)**. Purple: PI channel image **(E,G–I,K)**.

P56-P60 C57BL/6 male mice about 23 g were used in this study. For the virus injection, these mice were anesthetized with an intraperitoneal injection of 10% urethane and 2% chloralic hydras (0.1 ml per 100 g body weight). For single neuron tracing, we injected 100 nl of AAV-Cre virus and 100 nl of AAV-EF1α-DIO-EYFP virus [3.3 × 10^12^ genome copies (gc) ml^−1^] in the VPM nucleus at the same time. For sparse VPM neuron labeling, we tested the concentration of AAV-Cre from 1.27 × 10^9^ gc ml^−1^ to 1.27 × 10^12^ gc ml^−1^. 100 nl of AAV-Cre virus (1.27 × 10^12^ gc ml^−1^) infected extremely dense VPM neurons, and 1.27 × 10^11^ gc ml^−1^ about 200 neurons, and 1.27 × 10^10^ gc ml^−1^ about 80 neurons, and 1.27 × 10^9^ gc ml^−1^ about 15 neurons. Meanwhile, when the infection time was 2 weeks, not only the dendrites around soma were not clear, but also the axons. When the infection time was 30 days, the distal axons were bright in Figures 1J,K. Thus, the optimal titer of the AAV-Cre virus at the end is 100 nl, 1.27 × 10^9^ gc ml^−1^ to get this sparse labeling in Figure 1D. To get strongly labeled VPM neurons, we optimized the expression time of this dual-virus system in 18 mice for 30 days before brain specimen preparation. For projections of Sp5i neurons to the VPM nucleus, we injected 200 nl of CAV2-Cre virus in the VPM nucleus and 100 nl of AAV-EF1α-DIO-mCherry virus in contralateral Sp5i nucleus in the same mice. To get brightly labeled Sp5i axons, we optimized the expression time of this retro-virus system in 5 mice for 21 days, and found 21 days was suitable. All the viruses were injected by Nanoliter 2010 Injector (World Precision Instruments) stereotaxically and then delivered at a speed of 60 nl min^−1^. After the surgery, we stitched the incisions and then applied lincomycin hydrochloride and lidocaine hydrochloride gel to prevent inflammation and alleviate pain for the mice. The stereotaxic coordinates were VPM (A/P: −1.7 mm, M/L: −1.7 mm; D/V: −3.45 mm depth from pia surface), and Sp5i (A/P: −5.8 mm, M/L: −1.89 mm, D/V: −5.45 mm depth from pia surface), which was referred to the Allen Reference Atlas. All viruses were bought from the UNC Vector Core (Chapel Hill, North Carolina). All experiments and animal care were approved by the Administrative Panel on Laboratory Animal Care (APLAC) at Stanford University or the Institutional Animal Ethics Committee of Huazhong University of Science and Technology.

### Tissue Preparation

All labeled mice were deeply anesthetized by intraperitoneal injection of a solution containing 10% urethane and 2% chloralic hydras. Then, mice were perfused with 100 ml of 0.01 M PBS (Sigma-Aldrich) followed by 50 ml of 4% (w/v) paraformaldehyde (PFA) (Sigma-Aldrich) in PBS. The brains were removed from the skull and postfixed in 50 ml of 4% (w/v) PFA solution at 4°C for 24 h, and next immersed in 50 ml of 0.01 M PBS for 12 h twice.

### Whole-brain Embedding

For whole-brain imaging, the intact brain was resin-embedded. Firstly, intact brains were dehydrated via immersion in a graded ethanol series (2 h each at 4°C). After dehydration, we removed ethanol via 2 h immersions in 50, 75, 95% HM20 resin, and 100% HM20 resin (Electron Microscopy Sciences, cat. no. 14340) overnight at 4°C. Then, we infiltrated samples with 0.05 M Na_2_CO_3_ in HM20 resin at 4°C for 24 h. Subsequently, the impregnated samples were heat-polymerized and embedded in a vacuum oven at graded series of temperatures. Furthermore, a previous study described the details of the sample processing procedures (Gang et al., 2017; Guo et al., 2017).

### Brain Section Imaging

For whole morphology tracing experiments, the brains were cut consecutively into 100 μm-thick coronal sections on a vibratome microtome (Leica 1200S), and the coronal sections were gathered serially in PBS. The sections were incubated with 10 μg/mL (m/v) DAPI (Sigma-Aldrich) at room temperature for 10 min, and infiltrated 15 min in 0.01 M PBS three times repeatedly, and infiltrated in 50% glycerol (v/v), and then were mounted onto slides. The fluorescent samples were imaged with a 10×, 0.45 NA objective under a confocal microscope (Carl Zeiss, LSM710).

### Whole Brain CSFT Imaging

The well-embedded brain sample was fixed on a metal base of the chemical sectioning fluorescence tomography (CSFT) system (Wang et al., 2020). This system consisted of a green channel and propidium iodide (PI) channel (Li et al., 2010; Gong et al., 2016). A blue laser (488 nm) was used for excitation EYFP-tagged neuronal signals. PI stained the nucleic acids in the cell soma in the PI channel, revealing the soma and a portion of the dendrites and axon hillock to capture the whole brain cytoarchitecture. The embedded brains were immersed in 0.05 M Na_2_CO_3_ (v/v) solution for chemical reactivation to enhance well EYFP signals (Xiong et al., 2014). Moreover, a 60× water objective (NA 1.0) was immersed in this solution and used for imaging. This system used an afterward image montage with a 10 μm overlap and run in a stripe scanning mode (x axis) to realize large-scale imaging data acquisition (Yang et al., 2015). After the imaging parameters setting manually, the samples were sectioned in an antero-posterior (A-P) direction and imaged automatically at a voxel size of 0.2 × 0.2 × 1 μm to obtain the whole brain dataset.

### Brain Region Segmentation

**Figure 2:**
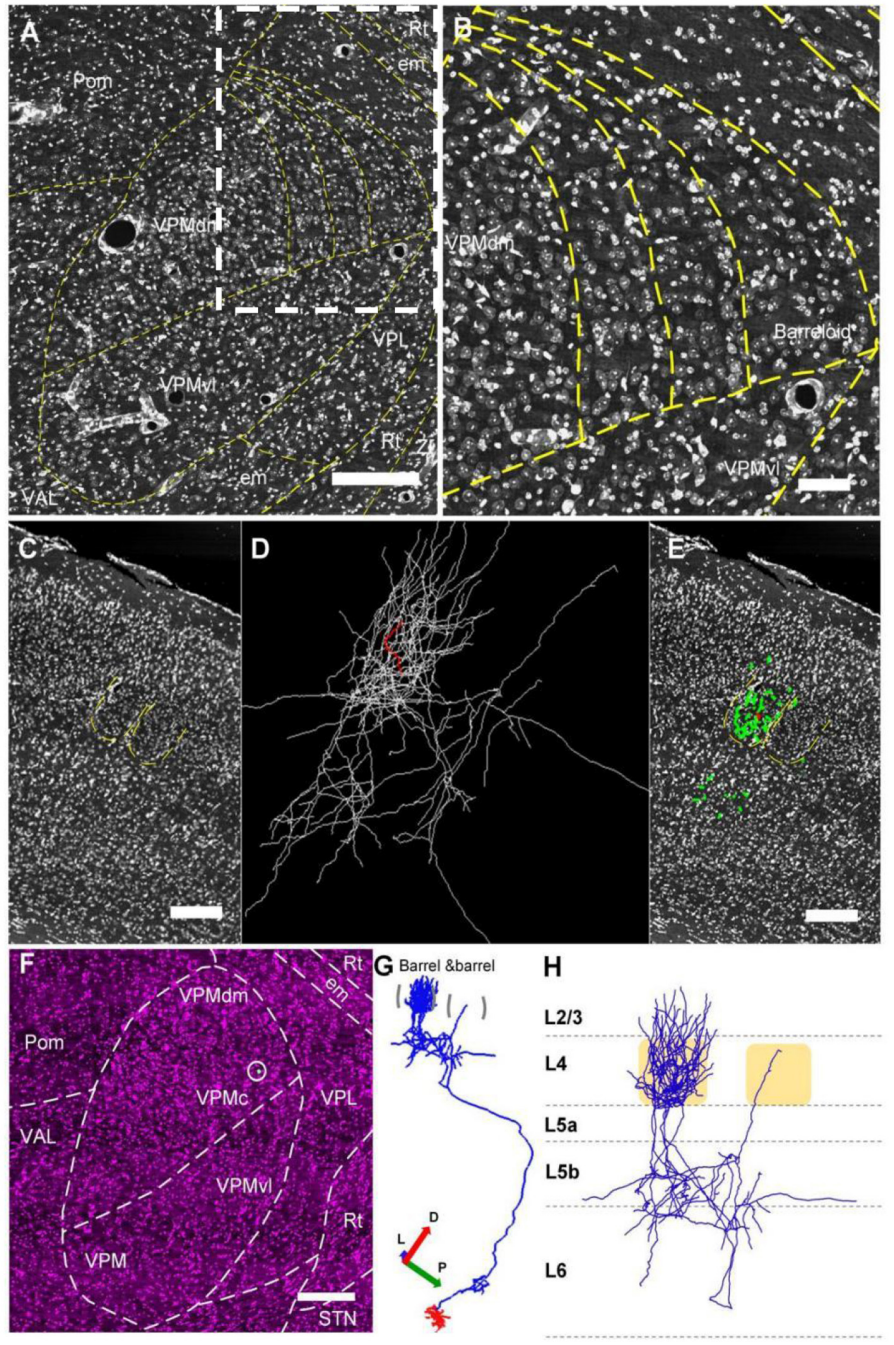
The whole reconstructed 3D morphology of an individual VPM neuron. **(A,B)** Constructing the border between VPMdm and VPMvl region. **(A)** Manually outlined contours of the borders of barreloids and the borders between the VPMdm and VPMvl compartments. **(B)** The amplification from the white dashed box in **(A)**. **(C–E)** The thalamocortical organization of an individual VPM neuron. This VPM neuron projects to the barrel column in cortex. Green and white lines in **(D)** and **(E)**: the same reconstructed morphology in cortex of VPM neuron. Red lines: axons in the barrel. Regions indicated by yellow in **(C)** and **(E)**: barrels and septa. **(F–H)** Whole reconstructed 3D morphology of this VPM neuron. **(F)** The VPM neuron in the VPMc region. White dot in white circle: soma. **(G)** The 3D morphology of VPMc neuron projecting to barrel column and Rt. Blue lines: axons. Red lines: dendrites. **(H)** Axon distribution in the barrel column. Scale bars, 250 μm **(A–H)**. D, Dorsal; P, Posterior; L, Lateral.

**Figure 3:**
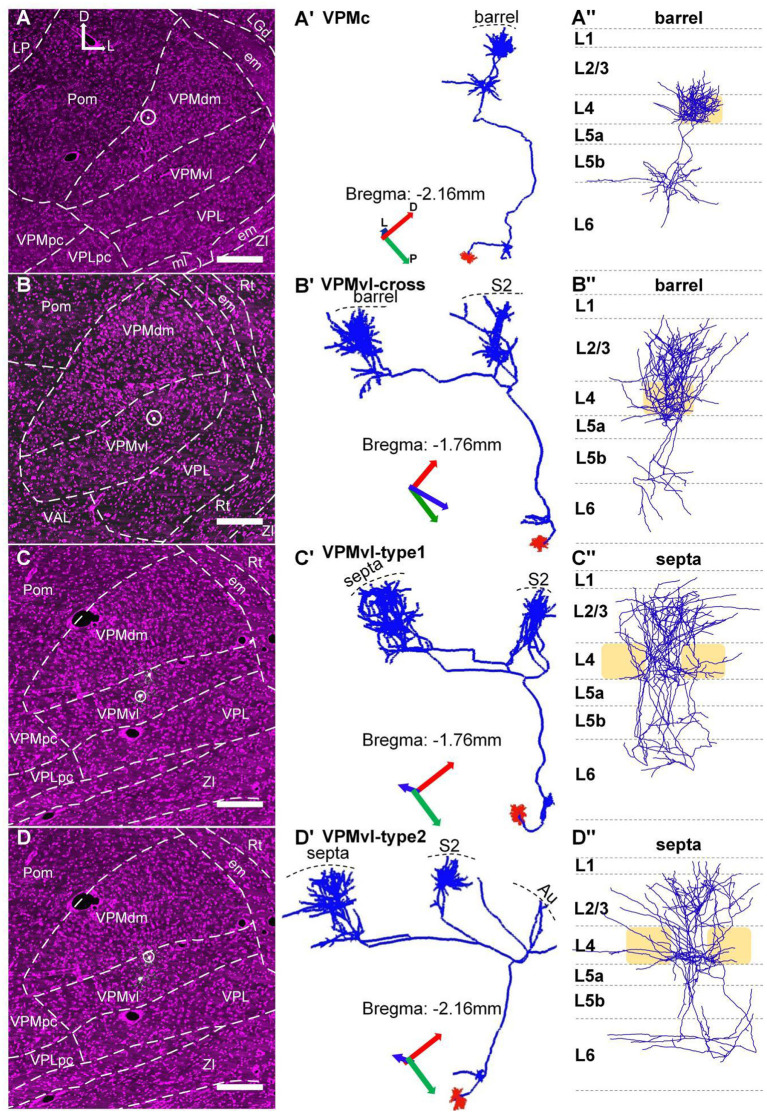
Topographic organization of VPM thalamocortical projections. **(A–A”)** The thalamocortical projection area of the typical VPMc neuron is a barrel column. **(B–B”)** Thalamocortical projection areas of VPMvl-cross neuron are barrel column and S2. **(C–C”**) Thalamocortical projection areas of VPMvl-type1 neuron are septa and S2. **(D–D”)** Thalamocortical projection areas of VPMvl-type2 neuron are septa, S2, and Au. **(A–D)** The VPM neuron soma location. White dots in white circle: soma bodies. **(A'–D')** The whole 3D morphology of VPM neuron. All project to Rt nuclei in subcortex. Blue lines: axons. Red lines: dendrites. **(A”–D”)** Axon distribution in barrel columns and septal areas. Light yellow box: barrel. The previous report naming convention is adopted, which relates the projection pattern to the cell body location in VPM. D, Dorsal; P, Posterior; L, Lateral.

To define VPM soma locations and axon projections, we mapped the boundaries between brain regions manually. Because of big mouse TB-sized datasets, we downsampled the data of raw image from 0.2 × 0.2 × 1 μm to 0.6 × 0.6 × 10 μm or 2.0 × 2.0 × 10 μm or 4.0 × 4.0 × 10 μm at a voxel size. The appropriate cropping areas were extracted according to the calculated coordinates. We used a series of 5–20 μm max intensity projections of PI images to discriminate the layers in the cortex and the regions in the subcortex according to the difference of cell architecture based on the networked version of the Allen Mouse Brain Reference Atlas (http://atlas.brain-map.org). The L1/2 boundary was discriminated by extremely sparse cell bodies in L1. Because there is no clear L2/ L3 boundary in other areas except PFC, the L2/L3 border was not drawn. In our study, layer 2 was the upper half of L2/3, and layer 3 was the lower half. The L3/4 boundary and L4/5 boundary were discriminated by the appearance of dense cell bodies forming a distinct band in L4. The L5a/5b was discriminated based on the existence of large cell bodies in L5a. The L5/6 was discriminated based on the existence of dense and horizontal oriented cell bodies and the defect of large cell bodies in L6. The border between barrel and septa was discriminated by clear barrel architecture. The VPMdm region and VPMvl region boundary was discriminated by the dense cells and the barreloid arrangement in the VPMdm region by the fine micron-scale imaging in this study. There are some rod-shaped cell architectures with their long axes from dorsomedial to ventrolateral and a length gradient in VPMdm region, which are same as cytochrome oxidase (CO)-stained sections in previous studies (Urbain and Deschênes, 2007). We used 5 μm-max intensity projections of successive PI brain slices that were 1 μm in each layer, so that the dense cells and barreloid arrangement of VPMdm can be clearly displayed to distinguish VPMdm and VPMvl regions (Figures 2A,B). Moreover, the location of the reconstructed VPMvl-cross neurons we chose are far away from the border between VPMdm region and VPMvl region (Figure 3B). Thus, the new type of VPM projection are certainly in VPMvl region, not in so called middle strait (Pierret et al., 2000). The dense cell bodies determined the border between Pom and VPM in VPM. The sparse and large cell bodies determined the border between VPM and VPL in VPL. The clear band architecture of Rt discriminated the boundary between VPL and Rt. Then, we manually mapped these boundaries between these areas using Amira 5.4.0 software *(FEI, Me'rignac Cedex, France)*.

### Reconstruction for 3D Morphology and Registration

To reconstruct the morphology of individual VPM neurons from the whole brain image datasets up to 10 TB, we preprocessed the sectioned brain datasets from TIFF format raw images series to mostd format for the feasible image display (Yuxin et al., 2017). We identified the VPM neurons that need to be traced. We imported the processed datasets into NeuroGPSTree software and reconstructed the complete morphology of VPM neurons manually (Quan et al., 2016). A whole morphology of VPM neuron took up to nearly 3 days to complete. Tracking information of VPM neurons was stored in SWC format with original location data. To guarantee all reconstructions of VPM neurons to be correct and complete, we rechecked each initial reconstruction with the same software. The VPM neurons without completely reconstructed axon collateral were eliminated.

We registered three whole-brain datasets to a unified brain space to benefit data analysis. We determined the reference brain space using one of the three brains. We extracted a global rigid image registration based on contours and the fine local registration of symmetric diffeomorphic normalization base on Demons to release three-dimensional co-registration of the other two brains to the reference brain. Subsequently, we registered the SWC files of neuron reconstructions in three brains to the reference brain using the registration parameters.

### Data Analysis

#### Length Density Analysis of VPM Axons in Cortex

We used custom MATLAB routines to analyze the length density of VPM thalamocortical axons. We obtained each orientation-readjusted VPM reconstruction in the cortex using the previous method (Akram and Wang, 2019). We presented the VPM thalamocortical distribution patterns (i.e., xy plane) in the heatmap. Then we presented the length density profile of VPM clusters along the cortical depth (just y axis) through integrating axonal length from this heatmap. We normalized profiles by dividing the fibers total length of the cells (length ratio). We also plotted layer boundaries in the length density figures (dashed lines). The positions in the y axis of layer boundaries were the average coordinates of all the contouring points covering the range of neuron fibers in x direction (Akram and Wang, 2019). Meanwhile, we normalized the laminar distribution of VPM axonal arbors to a criterion cortex template (just y axis) for comparative analysis among VPM neurons in different brain regions.

To address the difference of the thickness of the same layer in different cortical regions, we achieved standardization of the thickness of each layer for matching unnormalized analysis of length density (Johnson and Sinanovic, 2001; Akram and Wang, 2019). Then we standardized length density distribution curves of the VPM clusters for every neuron. The axon length was standardized as [axon length] / [the full length for a certain cluster] for comparing different cells.

#### Length Quantitative Analysis of VPM Axons in Cortex

Also, we made the quantification of the length, branches, and the volume of the soma about the reconstructed VPM morphology with the L-Measure software (Scorcioni et al., 2008). The height and width of VPM clusters in the cortex were measured using the Amira 5.4.0 software. All quantitative data are listed with the mean defined as ± s.e.m. Then the quantitative analyses of these data were performed using the GraphPad Prism software about multiple comparison tests after one-way analyses of variance (ANOVA).

## Results

### A Strategy for Achieving Complete Individual Neurons of VPM

To obtain a single complete morphology of VPM neurons, we devised a strategy comprising three portions, including virus labeling, whole-brain imaging, and neuronal reconstruction (Figure 1B). Firstly, a dual-virus system was applied to label individual neurons in their entirety (Figures 1A,B). AAV-EF1α-DIO-EYFP and AAV-Cre were injected in VPM nuclei for EYFP expression of VPM neurons. We optimized a dual-virus labeling system, namely reducing AAV-Cre virus titer and extending the time of virus expression for labeling VPM neurons sparsely and brightly (see Methods). We finally chose 1.2 × 10^9^ gc ml^−1^ of AAV-Cre virus and 30 days for virus expression through the gradient test. Secondly, the embedded brains with fluorescence signals were sectioned and imaged automatically with a powerful CSFT system to obtain high-resolution datasets in order to achieve long-term axon in the whole brain (Figure 1B2 and Supplementary Figures S1A,B). Thirdly, we reconstructed the entire morphology of VPM neurons after obtaining a single complete information dataset through facilitating the precise tracing of long-range projections (Yang et al., 2015; Gong et al., 2016) (Figure 1B3).

Through 3D datasets of a whole mouse brain, we got 8 labeled VPM neurons in VPMdm and VPMvl (Figures 1C,D and Supplementary Figures S1C–F). Moreover, we got explicit remote dendrites and spines (Figure 1F) and some characteristics of distal VPM axons, including terminal boutons, and axon branches (Figure 1K), benefiting from our studied strategy of the sparse and bright dual-virus labeling system and fine CSFT system. The overall projections of these VPM neurons enter reticular nuclei (Rt), barrel columns, septa areas, and S2 as previously reported (Pierret et al., 2000; Bokor et al., 2005) (Figures 1C,G,H and Supplementary Movies S1). Meanwhile, we also found some VPM neurons projected to the auditory cortex (Au) (Figure 1I and Supplementary Movies S1). VPM axons in S1BF spread from layer 6 to layer 1 forming a characteristic dense plexus in layer 4 (Jensen and Killackey, 1987; Bokor et al., 2005) (Figure 1G). Axons in S2 spread from layer 6 to layer 2 (Figure 1H). We also found axons in Au spread from layer 6 to layer 4 (Figure 1I). VPM dendrites are rich and have closely packed dendritic excrescences, similar to what was previously reported (Ohara et al., 1995) (Figure 1E).

In order to find some fine long-range projections, we performed the whole morphological reconstruction of individual VPM neurons. Firstly, we clearly identified VPM region from the surrounding brain regions, and the VPMdm and VPMvl regions based on the cytoarchitecture of the barreloids in the VPMdm region (Figures 2A,B). We reconstructed a VPMc neuron based on fiber tracing, cytoarchitecture of brain regions, and soma localization (Figures 2C–F), and found that the axon distribution of this VPMc neuron is as in the previous report (Meyer et al., 2010; Oberlaender et al., 2012). This VPMc neuron innervates Rt and the barrel columns (Figure 2G). Meanwhile, the axons in the barrel column have a packed plexus in layer 4 and spread from layer 6 to layer 3 (Figure 2H).

### The Diversity of Thalamocortical Projection Patterns of VPM Neurons

**Figure 4:**
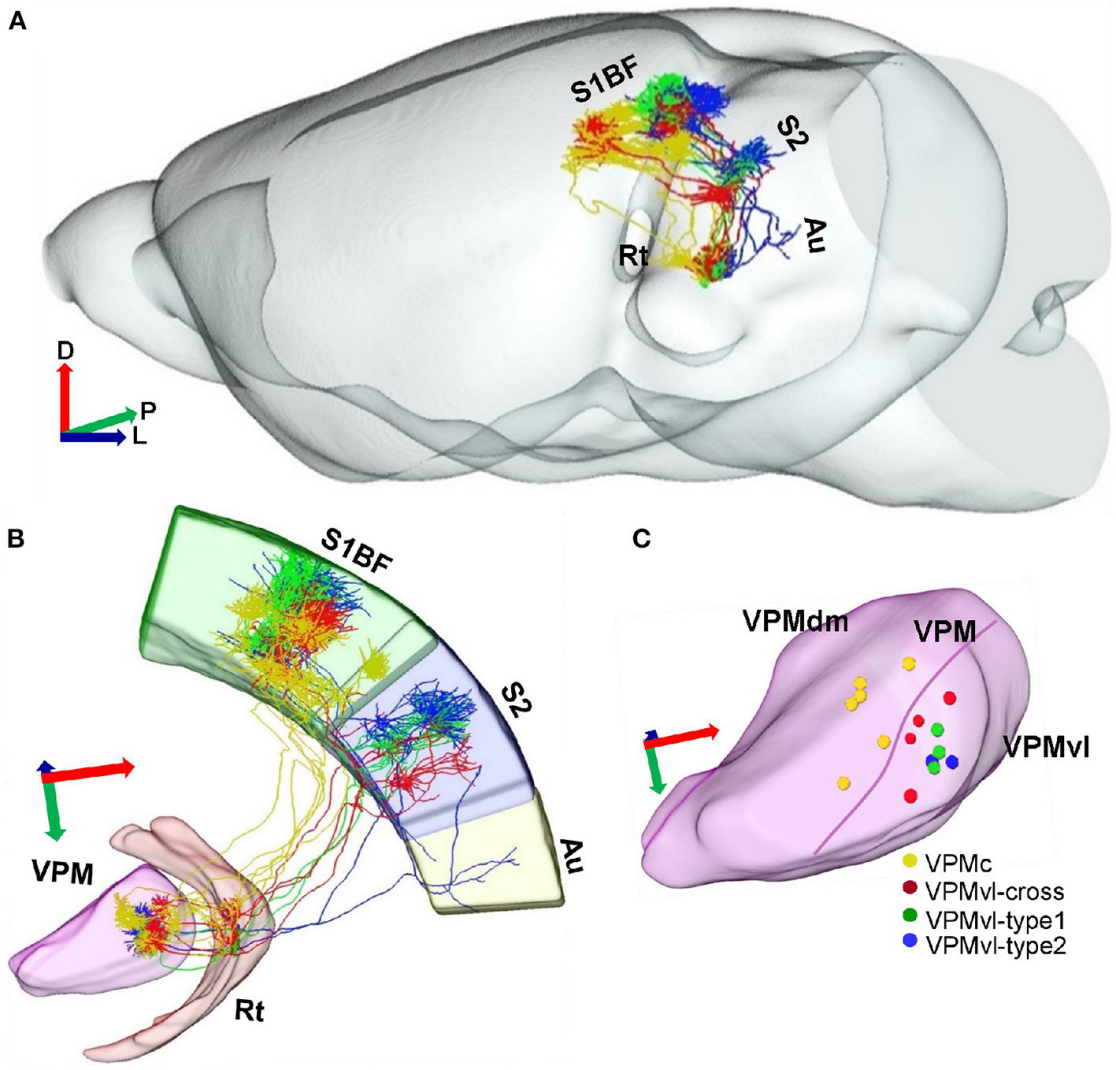
Projection organization of four different VPM subgroups. **(A)** Overlay of 15 reconstructed VPM neurons registered into a reference brain. Gray represents the outer contour of the mouse brain. Yellow lines indicate VPMc morphologies. Green lines indicate VPMvl-cross morphologies. Red lines indicate VPMvl-type1 morphologies. Blue lines indicate VPMvl-type2 morphologies. **(B)** Fifteen reconstructed VPM neurons and fiber distribution. **(C)** The soma localization of four subgroups in different VPM subregions. D, Dorsal; P, Posterior; L, Lateral.

We achieved 15 whole morphologies of VPM neurons from three brains. Those reconstructed neurons are registered into a reference brain (Figure 4A). We classified those reconstructed neurons into four types, including VPMc, VPMvl-type1, VPMvl-type2, and VPMvl-cross, basing on thalamocortical projection patterns and soma location (Figures 3A–D,A'–D'). The five typical VPMc neurons project to barrel columns from layer 6 to layer 3, consistent with the previous study (Figures 3A–A”, Figure 4, Supplemental Figure S2, and Supplemental Figure S3A). The 9 VPMvl neurons are diverse in patterns of thalamocortical axons. Among these, we found surprisingly the 4 VPMvl neurons mainly project to barrel columns from layer 6 to layer 2, and S2 from layer 6 to layer 2, which we named VPMvl-cross as a new VPM type (Figures 3B–B”, Figure 4 and Supplementary Figure S3B). The three typical VPMvl-type1 neurons project to septal areas from layer 6 to layer 1, and S2 from layer 6 to layer 2 (Figures 3C–C”, Figure 4 and Supplementary Figure S3C). The 2 VPMvl-type2 neurons project to septal areas from layer 6 to layer 1 and S2 from layer 6 to layer 2 and Au with sparse fibers (Figures 3D–D”, Figure 4 and Supplementary Figure S3D). Therefore, the thalamocortical projections of VPMvl-cross neurons constitute the outputs to barrel columns, which is the first reported cross-streams from VPMvl to barrel columns between the main two parallel pathways to our best knowledge.

### The Morphological Properties of the Different VPM Subgroups

**Figure 5:**
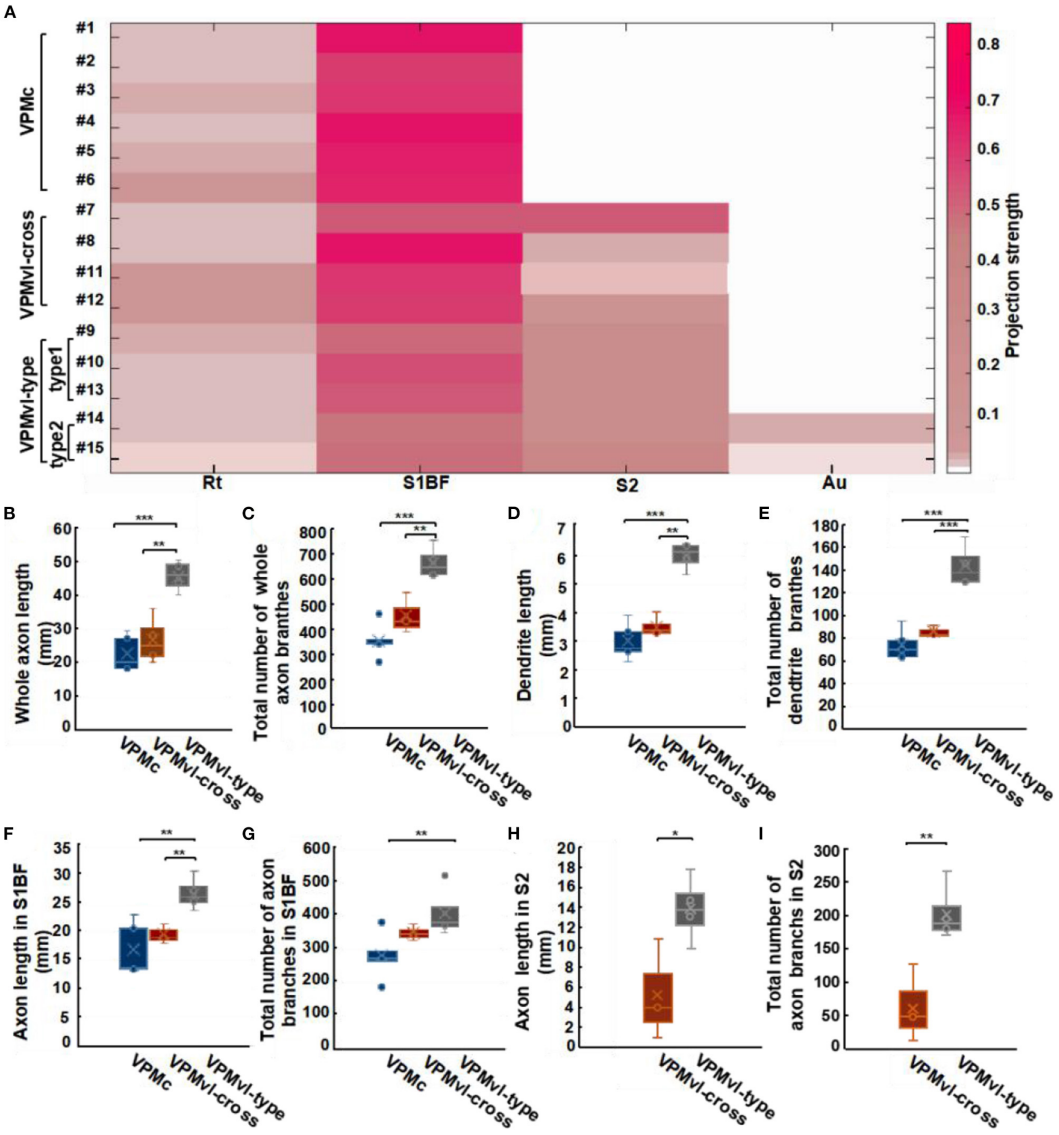
Characteristic analysis of three VPM subgroups in different areas. **(A)** Projection strengths in different regions in target areas for each VPM neuron. Comparisons of whole axonal length **(B)** and the total number of axonal branches **(C)** among VPMc, VPMvl-cross, and VPMvl-type. Comparisons of dendritic length **(D)** and the total number of dendritic branches **(E)**. Comparisons of axon length in S1BF **(F)** and the total number of axon branches in S1BF **(G)**. Comparisons of axon length in S2 **(H)**, the total number of axon branches in S2 **(I)** between the VPMvl-cross and the VPMvl-type. VPMc: *n* = 6; VPMvl-cross: *n* = 4; VPMvl-type (VPMvl-type1 and VPMvl-type2): *n* = 4. *p < 0.05, **p < 0.01, ***p < 0.001, n.s. Represents no significant correlations.

There are few fibers in Au from VPMvl-type2 neurons, we classify VPMvl-type1 and VPMvl-type2 into a category and collectively name VPMvl-type in order for comparative analysis. The results showed that these three VPM subgroups, VPMc, VPMvl-type, and VPMvl-cross, displayed different projection strengths in their target areas (Figure 5A). We made a quantitative comparison on the soma volume and the length and the total number of branches of axons and dendrites (Supplementary Table 1). We found that VPMvl-cross neurons and VPMc neurons have no statistical difference in the soma volume, the length, and the total number of branches of dendrites and axons in barrel columns, S1BF, Rt, and in the whole cortex (Figures 5B–G and Supplementary Figures S4A–E). Nevertheless, the length and the total number of branches of dendrites and axons in S1BF, S2, and in the whole cortex of VPMvl-cross neurons are much less than those of the VPMvl-type neurons (Figures 5H,I and Supplementary Figures S4C,D). These results implied that VPMvl-cross neurons have similar inputs and outputs to VPMc neurons in barrel columns, S1BF, Rt, and in the whole cortex, and have fewer inputs and outputs than VPMvl-type neurons in S1BF, S2, and in the whole cortex.

Meanwhile, the three VPM subgroups preferentially project to the cortex rather than to the subcortex, showing that these neurons have more innervation to the cortex in the whole brain (Supplementary Figure S4F). The VPMvl-cross and VPMvl-type neurons preferentially project to S1BF rather than S2, showing that these neurons have more innervation to S1BF in the cortex (Supplementary Figure S4G). These results indicate that VPM neurons have projection preferences for the cortex in the whole brain and projection preferences for the S1BF region in the cortex.

### Distribution Patterns in Layers of Thalamocortical Axons of the Three VPM Subgroups

In previous studies, axonal length is linear fits the number of boutons, allowing estimate strength of synaptic connectivity (Peters, 1979; Oberlaender et al., 2012). In order to compare the projection strength of VPM neurons within different layers in the cortex, we calculated axon density distribution in detail and collapsed the densities to 1D synapse profiles along the cortical depth.

**Figure 6:**
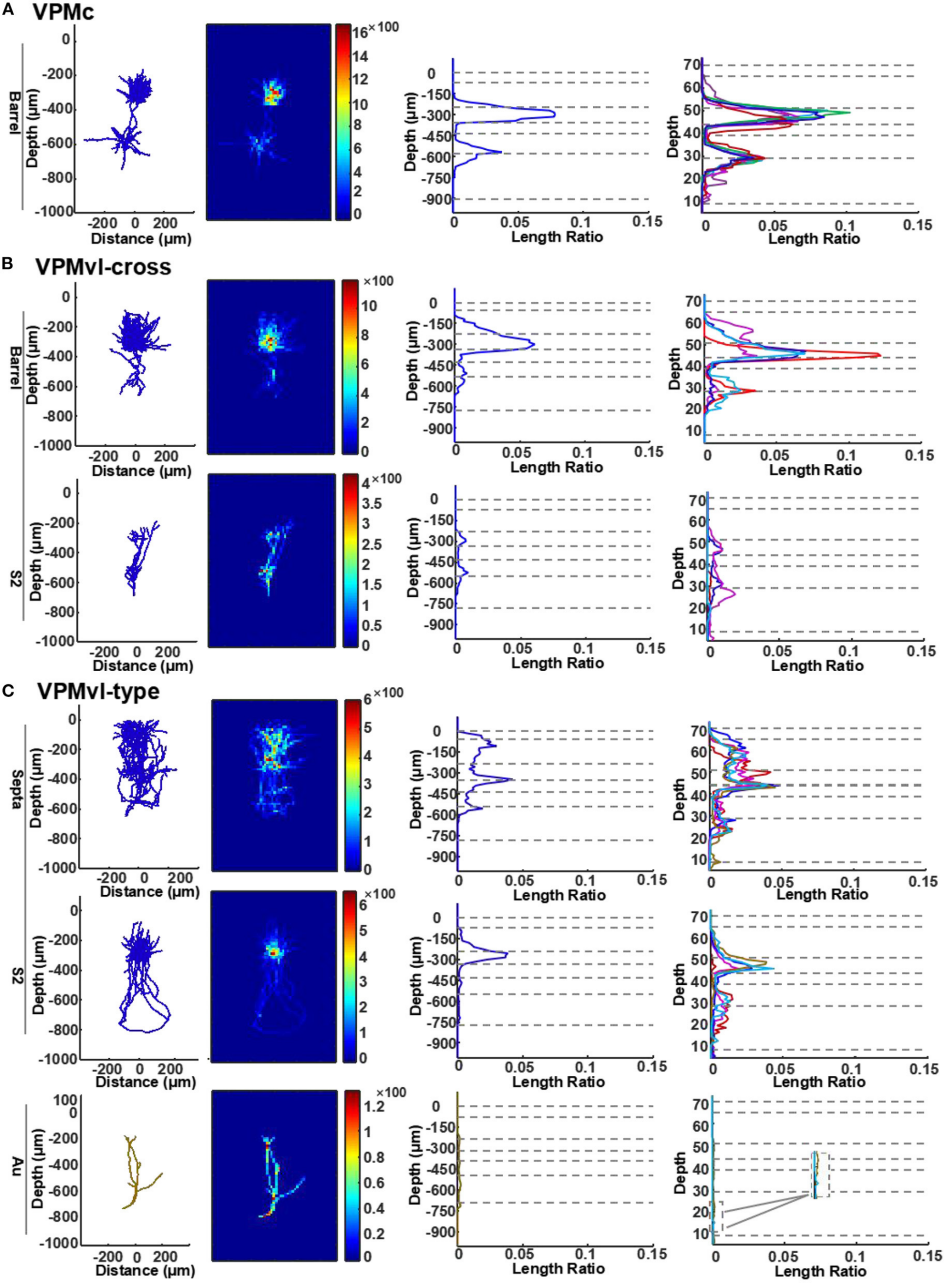
Quantitative analysis of VPM axons in different layers of the cortex. **(A)** Length density analysis of thalamocortical axons in different layers of VPMc neurons. For typical VPMc neurons, the row is for axons in barrel columns. The first column: projection of reconstructions (axons in blue). The second column: heatmap of length density distribution of axons. The third column: length density plots of axons along cortical depth (y axis). The forth column: normalized density plots of axons along cortical depth, including axons of the remaining VPM neurons (see more details in STAR Methods). Dashed horizontal lines represent cortical layer borders. Different colored lines represent different cells. VPMc: *n* = 6. **(B)** Length density analysis of thalamocortical axons in different layers of VPMvl-cross neurons. For VPM-cross neurons, the above row is for axons in barrel columns, the bottom for axons in S2. VPMvl-cross: *n* = 4. **(C)** Length density analysis of thalamocortical axons in different layers of VPMvl-type neurons. For VPMvl-type neurons, the above row is for typical axons in septa, the middle for typical axons in S2, and also the bottom for axons in Au of the other two VPMvl-type2 neurons. Inset: enlarged from the box, which highlights axon branches in deep layers in Au region. Ratio presents density value. VPMvl-type (VPMvl-type1 and VPMvl-type2): *n* = 4.

Firstly, the profiles in barrel columns of the VPMc neurons display two peaks, the big one in layer 4 and the small one at the border between layer 5 and layer 6 (Figure 6A and Supplementary Figure S5A). These results indicate that VPMc neurons have strong projective intensity in layer 4 and the region between layer 5 and layer 6. Secondly, the profiles in barrel columns of the VPMvl-cross neurons are similar to those of the VPMc neurons. Additionally, the profiles in S2 of the VPMvl-cross neurons have two peaks, one in layer 4 and another at the border between layer 5b and layer 6 (Figure 6B and Supplementary Figure S5B). The projection density of VPMvl-cross neurons in layer 4 of barrel columns is higher than that in S2. Thirdly, the profiles in septa from the VPMvl-type neurons displayed three peaks, the biggest one in layer 4 and the other two in layer 6 and in layer 2/3. In addition, the profiles in S2 had two peaks, in layer 4 and at the border between layer 5b and layer 6. However, the profiles in Au had no evident peak (Figure 6C and Supplementary Figures S5C,D). These results show that VPMvl-cross neurons and the VPMc neurons have similar patterns of axon distribution in S1BF along the vertical axis.

### The Projections of Sp5i Neurons to VPM Subregions

**Figure 7:**
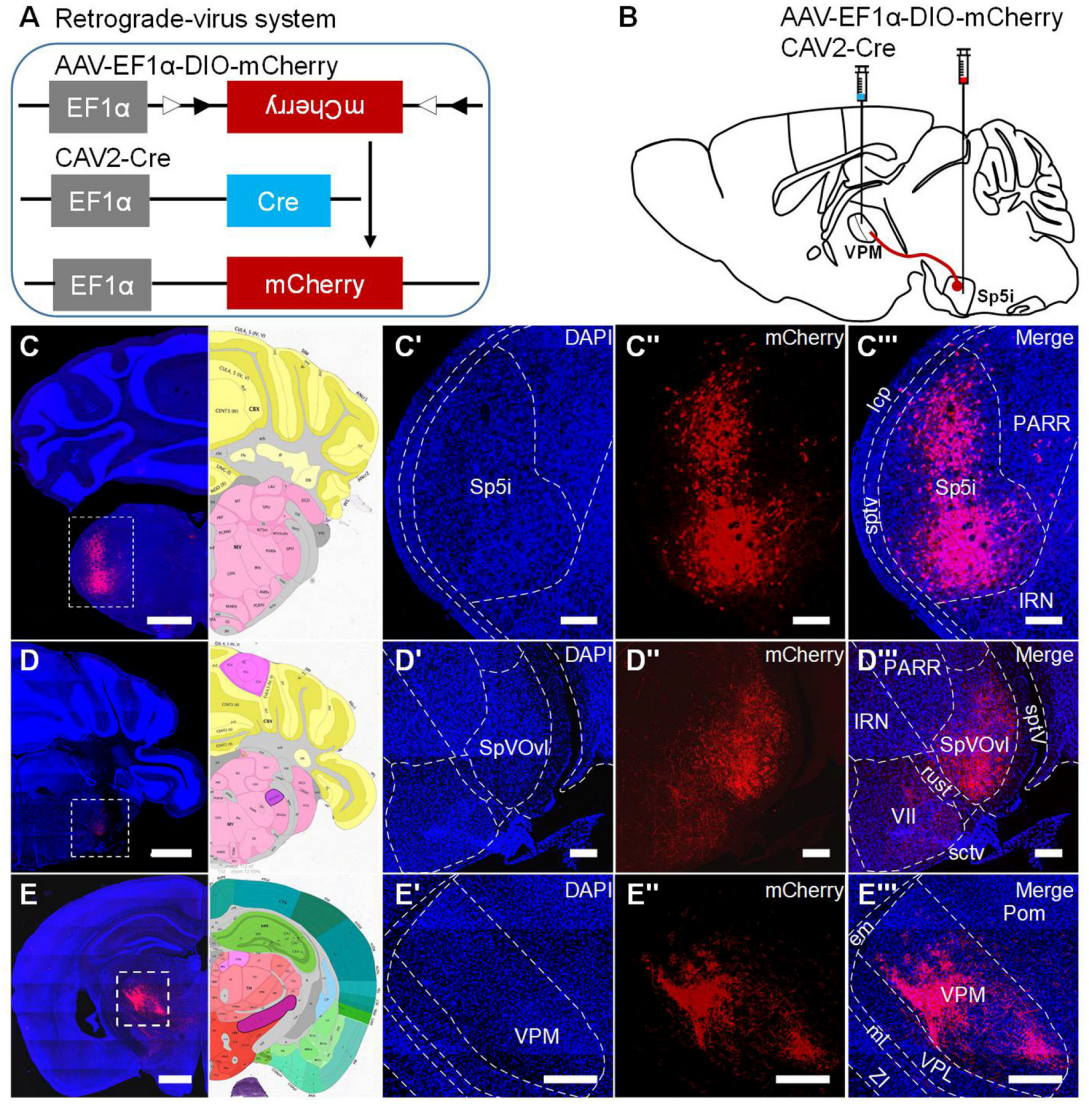
Inputs from Sp5i neurons to VPMdm region. **(A)** Schematic diagram showing the experimental processes to generate mCherry protein. **(B)** Virus injection paradigm for labeling Sp5i neurons. The retrograde CAV2-Cre virus was injected into VPM. AAV-EF1α-DIO-mCherry virus was injected into Sp5i on the contralateral side. **(C–C”')** Sp5i neurons were labeled. **(C)** The coronal section was having infected Sp5i neurons and its corresponding brain map. Amplification of DAPI channel image **(C')** and mCherry channel image **(C”)** and merged channel image with diagram of Sp5i nuclei **(C”')** from the white dashed box in **(C)**. **(D–D”')** Sp5i axons project to SpVOvl between Sp5i and Pr5 without neurons infected. **(E–E”')** Sp5i axons covering most of VPM region including VPMvl region and VPMdm region. Dashed lines: borders between regions. Scale bars: 1,000 μm **(C–E);** 250 μm **(C'–C”',D'–D”',E'–E”')**.

To determine whether there are direct inputs to VPMdm (VPMh and VPMc) region innervated by Sp5i neurons in the vibrissa sensory system, we studied the projection of Sp5i neurons through the retrograde-virus labeling system (Figures 7A,B). Plenty of Sp5i neurons were labeled as expected (Figures 7C–C”'). Sp5i neurons project abundantly to Sp5Ovl nuclei. There are no neurons labeled in Sp5Ovl nuclei (Figures 7D–D”'). Because Sp5Ovl nuclei is between Sp5i nuclei and Pr5 nuclei along the anteroposterior dimension. Therefore, the labeled neurons are concentrated in Sp5i nuclei. Surprisingly, the axons of Sp5i neurons project into not only the VPMvl region but also the VPMdm region sufficiently and brightly (Figures 7E–E”'). As previously reported, we found the axons of Sp5i neurons exist in the Pom region, while we found the axons of Sp5i neurons in the VPMdm region are much more than ones in Pom (Figure 7E”'). Our results show that the Sp5i neurons innervate both the VPMvl region and VPMdm region. Therefore, the trigeminothalamic projections of Sp5i neurons constitute the inputs of VPMdm, which is the first reported cross-streams from Sp5i neurons to VPMdm between the main two parallel pathways to our best knowledge.

## Discussion

**Figure 8:**
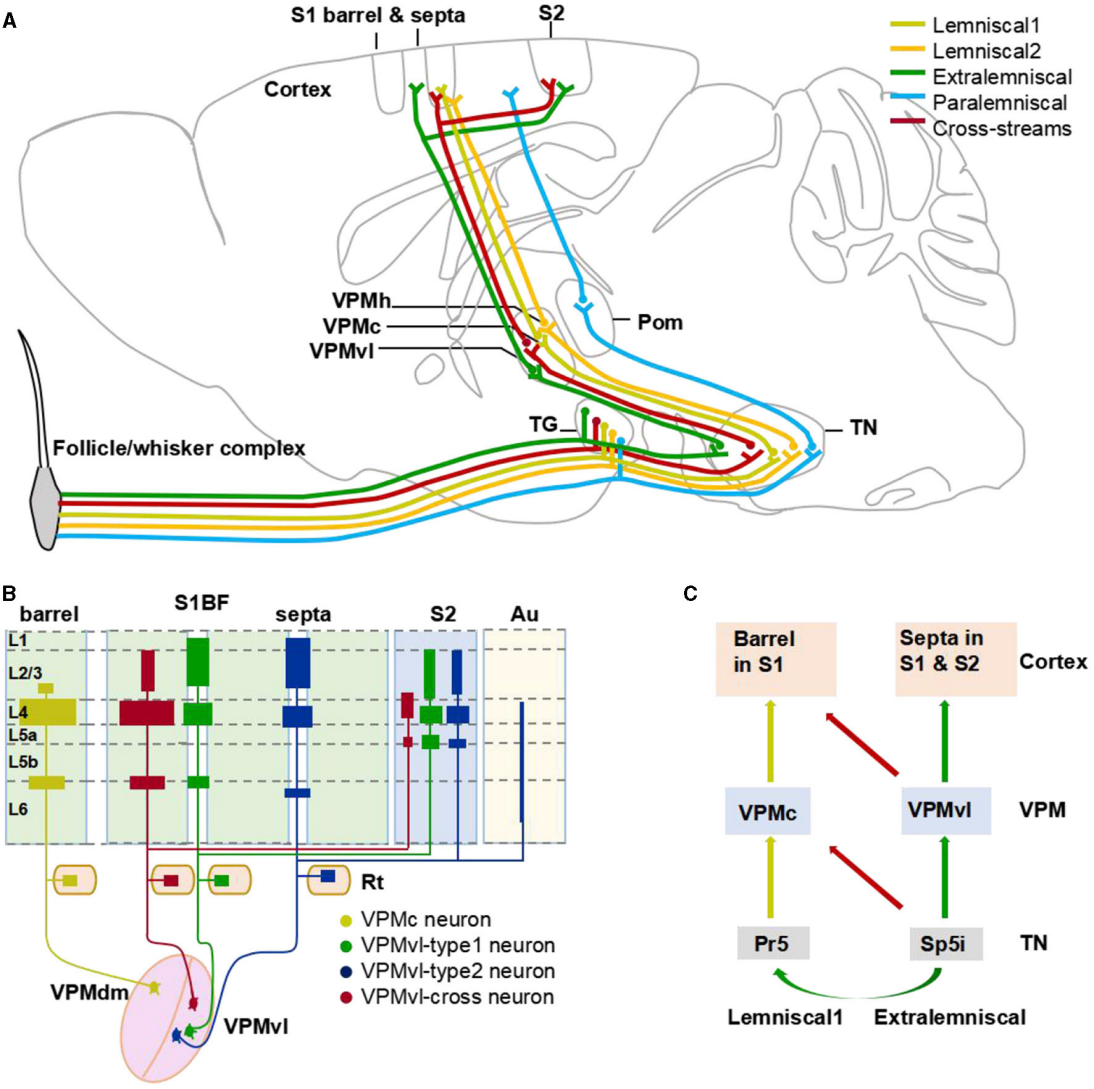
The cross-streams enrich whisker system. **(A)** An updated circuitry model among the brainstem, VPM, and barrel cortex. The circuits revealed in this study are shown in red color, and the conventional circuits are shown in other colors. Brown lines represent the lemniscal1 pathway. **(B)** Schematic diagram illustrating the major thalamocortical axon distribution of organization of VPM neurons. **(C)** A model of cross-streams between lemniscal1 pathway and extralemniscal pathway.

In this study, we identified the new anatomical cross-streams in VPM, including the outputs of VPMvl neurons projecting to barrel columns, and the inputs of VPMdm received from Sp5i (Figure 8). These results provide the missing pieces of anatomical evidence for subcortical pathways that might support multi-whisker integration in barrel columns. The new anatomical pathways ensure that vibrissal sensory signals are conveyed to implement more possibilities for whisker function and allow the mice to navigate their surroundings better. Furthermore, through sparse highlighting labeling and long-range fine imaging and tracing, we reconstructed the whole morphology of individual VPM neurons. Then, basing on soma position and thalamocortical projection patterns of complete VPM morphologies, we classified three subgroups of the VPM neurons, namely VPMc, VPMvl-cross, and VPMvl-type. In length and number of branches of both axons and dendrites, and in distribution densities of axons in cortical layers, the VPMvl-cross neurons and typical VPMc are close to each other, which meaning that they have many similar characteristics in input and output in the whole brain, and innervation patterns in different SIBF layers.

## Methodological Considerations

Many previous reports used various labeling methods to study the projection of VPM into the barrel cortex. For example, Saporta and Kruger's study showed the possibility that VPMvl neurons ascend directly to barrel columns by horseradish peroxidase retrogradely (Saporta and Kruger, 1977). However, this retrograde tracer was not restricted to inject a single barrel, which rendered this result doubtful and need further research (Pierret et al., 2000). Moreover, to investigate how VPM innervates the barrel cortex, Pierret used retrograde Fluoro-Gold to study the thalamocortical projection of VPM neurons (Pierret et al., 2000). However, the neurons were extremely sparsely labeled, the remote axons could not be reconstructed completely. The labeling strategies used in the above studies have apparent disadvantages, such as incomplete staining of axons and many signals being too weak (Mesulam and Rosene, 1977; Sellés-Navarro et al., 1996). In contrast, the current rapidly developing virus strategies have many advantages, such as labeled neurons having longer survival time and dilution of viruses that could be controllable for different labeling numbers (Betley and Sternson, 2011). Multiple innovative virus strategies combine with the imaging technology with rapid development and make it possible for us to track the circuits of the nervous system reliably. In order to reliably identify projections of VPM neurons to barrel cortex, sparse highlighting labeling and long-range tracing of VPM neurons are required. To realize sparse highlighting labeling of VPM neurons, we chose and optimized dual-virus labeling strategy namely reducing virus titer and extending the time of virus expression. Meanwhile, the data obtained by CSFT provides the information of cell cytoarchitecture to accurate locating of the nucleus. Based on these technologies, we reconstructed complete morphologies of individual VPM neurons and accurately located the VPM nucleus and the regions projected by VPM.

In this study, it is not easy to obtain the dataset of the completely reconstructed VPM neurons. It takes one month to satisfy the bright and sparse viral labeling of neurons in mice, 7 days for whole-brain embedding, 8 days for embedding sample slicing and signal acquisition, and 5 days for complete reconstruction of each neuron. Thus, there were only 15 reconstructed VPM neurons completely. While, it was sufficient to classify the reconstructed VPM neurons based on the complete reconstruction and accurate cytoarchitecture information. Thus, these results revealed the outputs of VPMvl neurons to barrel columns. Similarly, our results demonstrated the input of Sp5i neurons to VPMdm brain region.

### The Subcortical Pathways of Multi-whiskers Integration in Barrel Columns

The neuronal mechanisms underlying the sensory information conveying and processing can provide crucial knowledge to neuroscientists investigating how other neurosystems process and respond to specific inputs and the field of biomimetics, which focuses on developing biologically-inspired artificial systems (Diamond et al., 2008). A crucial question for understanding how sensory signal processing occurs is how multi-whisker information converges in the barrel columns (Fox et al., 2003). This issue concerns the way in which delicate sensory processing occurs in the barrel cortex (Fox et al., 2003). Moreover, it is necessary to know whether plasticity of surround receptive fields in the barrel is attributing to plasticity in cortical or subcortical pathways to discover the underlying plasticity mechanisms (Fox, 1994; Glazewski and Fox, 1996; Buonomano and Merzenich, 1998; Fox et al., 2003).

Multi-whisker integration in barrel neurons maybe generated minorly by sparse intracortical trans-columnar connections (Simons and Woolsey, 1979; Kwegyir-Afful et al., 2005). Subsequent studies found that multi-whisker signals in the barrel columns are determined primarily by direct inputs from VPM mediated by the intersubnuclear pathway from Sp5i to Pr5 in the subcortical pathway (Timofeeva et al., 2004; Kwegyir-Afful et al., 2005). The VPMh neurons projecting to septal areas also distribute 25% of their terminals in barrel columns, which may enable barrel columns to have multi-whisker receptive fields (Armstrong-James and Callahan, 1991; Friedberg et al., 1999; Furuta et al., 2009). Additionally, a large amount of the subcortical projections to the barrel cortex originate from the posterior medial nucleus of the thalamus (POm) with dense axonal arborizations in L1 and L5a (Meyer et al., 2010; Wimmer et al., 2010), which are also multi-whisker and have some overlapping projecting with the VPMvl-cross type neurons (Pierret et al., 2000; Timofeeva et al., 2004). However, these projections have no contribution to integrating multi-whiskers to barrel cortex due to the feed-forward inhibition from the zona incerta (ZI) to Pom (Trageser and Keller, 2004; Furuta et al., 2009; Lavallée et al., 2015).

Our research benefits from our sparse and bright labeling and fine imaging and reconstructing and locating in the present study. Our results revealed the outputs of VPMvl-cross neurons projecting to barrel columns and the inputs of VPMc neurons received from Sp5i. In addition, through quantitative measurements, we demonstrate that the clusters in S1BF of VPMvl-type neurons range from 557 to 634 μm along anteroposterior, and from 237 to 400 μm in the mediolateral dimension (Supplementary Table S2). However, the mice barrel column diameter is ~200 μm and is far wider than that of the septal region (Rachel, 2007). Thus, this means that these axonal arborization of VPMvl-type neurons are out of range of a single septal region and necessarily spread into surrounding barrel columns. Our reconstruction data showed that the axonal terminals of VPMvl-type neurons project to layer 2/3 of barrel columns (Supplementary Figure S3C). So, it seems that the VPMvl-type neurons might involve integrating multi-whiskers in layer 2/3 in surround barrel columns. Meanwhile, we also found certain VPMc neurons innervating several barrel columns simultaneously (Supplementary Figure S2A).

Briefly, the newly found cross-streams between the two main typical parallel pathways are including the main projection from VPMvl neurons to barrel columns and the large projection from Sp5i neurons to VPMdm region. Thus, these new circuits determine the ascending projections from the VPMvl region to the single barrel column, which were once due to not being restricted to the size of a single barrel by retrograde horseradish peroxidase retrograde tracing (Saporta and Kruger, 1977; Pierret et al., 2000). Also these new circuits identify that VPM neurons accept projections from both Pr5 and Sp5i simultaneously, and determine the precise location of these VPM neurons in the VPMdm region (Wang and Ohara, 1993; Friedberg et al., 1999; Pierret et al., 2000). Meanwhile, these cross-streams might explain the phenomenon that there are the residual adjacent whisker responses in barrel neurons from control animals showing a midline lesion (Kwegyir-Afful et al., 2005). Therefore, these cross-streams might be the subcortically ascending pathways to generate multi-whisker receptive fields of barrel columns to involve the execution of multi-whisker integration to encode spatio-temporal correlation patterns and improve the overall signal strength, and underlie directional tuning, and responds to context-dependent needs (Minnery et al., 2003; Diamond et al., 2008; Feldmeyer et al., 2013; Estebanez et al., 2016). Future experiments are needed to determine the functions of these new pathways on the multiwhisker-receptive fields of the barrel columns by electrophysiology recordings.

### Analysis of the Axon Projection in Whole Brain of the Different VPM Subgroups

Based on our dataset, we reconstructed uninterrupted complete morphologies of 15 VPM neurons. Our results showed that VPMvl-type2 neurons have extremely a few fibers in auditory cortex, which is first reported, might have a regulating effect on auditory function (Figures 3D, 5A and Supplementary Figure S3D). Moreover, the 5# neuron in VPMc region mainly innervating the septal columns has the identical projections of the VPMh cells (Desilets-Roy et al., 2002; Urbain and Deschênes, 2007) (Supplementary Figure S3A). This result indicates that this type of neurons not only locate in the VPMh region, but also distribute in the VPMc region. Therefore, the projections of some VPMc neurons might be the organization to support the multi-whisker responses of the barrel columns by subcortically ascending pathway.

Though every one type of VPM neurons had the same projection regions, the projection strengths are different (Figure 5A). The results indicated that each of these neurons projecting the same areas possess different innervation strengths. The axonal length of VPMvl-cross neuron was ~51 mm, which is by far the longest reported VPM axonal length in mouse (Figure 5B). All reconstructed VPM neurons (15 cells) project to Rt is consistent with the previous study with two neuronal morphologies (Oda et al., 2004) (Figures 3A'–D', 5A). And about 2.8–9.5% of VPM axons give off collaterals innervating RT with short and simple branches (Figure 5A). This result would seem to disagree with previous studies about 40% of VPM axons projecting into Rt in rat (16 cells) (Oda et al., 2004). It is important to note that the methods to obtain the morphology are significant differences, the experiments of previous studies used intracellular horseradish peroxidase injection technique (Harris, 1987). Rt cells project back to the VPMdm in a closed-loop pattern, and the Rt neurons are the source of inhibition in VPM (Desilets-Roy et al., 2002; Shepherd et al., 2005). VPMvl cells derive their receptive field from RT cells (Bokor et al., 2008). This result indicates that there also might exist a closed-loop pattern between VPMvl neurons and Rt neurons.

Meanwhile, the number of axonal branches in S1BF of VPMvl-cross neurons is close to those of VPMc neurons but much less than those of VPMvl-type neurons (Figure 5C). We know that there is a linear relationship between the number of branches and one of the branch points, and propagation of the action potential may slow down at the branch point (Debanne, 2004). Thus, the more axonal branches the neurons have, the lower the action potentials are delivered. Therefore, the VPMvl-cross cells and VPMc cells might evoke shorter axon terminal potential (AxTP) with latencies and need shorter conduction time than the VPMvl-type cells in S1BF, which need to be studied further by electrophysiological recordings.

### Different Innervation Patterns in Barrel Cortex of Different VPM Neuron Subgroups

With passive stimuli, the layers of barrel columns displayed functional differences, and some layers have the coding schemes of their thalamic counterparts, which might reflect tight thalamocortical processing (Ahissar and Zacksenhouse, 2001b; Derdikman et al., 2006). In barrel columns, layer 4 neurons that receive lemniscal1 afferents response for touch are similar to the neurons of the VPMdm region (Diamond, 1995; Pierret et al., 2000; Bureau et al., 2006). Meanwhile, layer 2/3 neurons that receive extralemniscal afferents respond for touch and whisking, similar to the neurons of the VPMvl neurons (Ahissar and Zacksenhouse, 2001a; Derdikman et al., 2006; Yu et al., 2006). It is reported that there was a linear relationship between the VPM axon distribution and the VPM bouton distribution in the rodent vibrissal cortex, and the latter reflects the strength of thalamocortical synaptic connectivity for cortex neurons (Oberlaender et al., 2012). In order to estimate the difference of strength of thalamocortical synaptic connectivity with different layers in barrel columns, we compared the axonal distribution densities in different layers of S1BF among different VPM neuron subgroups. The axonal distribution of VPMc neurons and VPMvl-cross neurons along the cortical depth in SIBF is similar but distinct from that of VPMvl-type neurons. The peaks of axon distribution of VPMc neurons and VPMvl-cross neurons appear in layer 4 and layer 5b/6a, while the peaks of VPMvl-type neurons are in layer 2/3, layer 4, and layer 6, the axonal densities of which are less than those of VPMc neurons and VPMvl-cross neurons. The results indicate that the innervation patterns in SIBF of VPMc neurons and VPMvl-cross neurons are different from those of VPMvl-type neurons, also suggest the first two have stronger synaptic connectivity with the layer 4 neurons than the latter. The distinct thalamocortical innervations in different layers of barrel cortex contributing to coding whisker information need to be studied further.

Finally, it was reported that layer 1 in the barrel cortex receives VPM fibers as well. Some of these axons are parallel to the brain surface. Nevertheless, the location of corresponding VPM neurons is not precise yet (Lu and Lin, 1993; Oda et al., 2004). Our results show that some axons of VPMvl neurons project to layer 1 and are parallel to the brain surface (Supplementary Figure S3C), which indicates that at least part of VPM fibers in layer 1 arises from the VPMvl region. Whether axons are originating from VPMdm neurons needs to be demonstrated further.

## Data Availability Statement

The original contributions presented in the study are included in the article/Supplementary Material, further inquiries can be directed to the corresponding authors.

## Ethics Statement

The animal study was reviewed and approved by Institutional Animal Ethics Committee of Huazhong University of Science and Technology.

## Author Contributions

TX, ZX, and SZ conceived and organized the study. HZ performed viral labeling and resin-embedding experiments, organized the confocal imaging, reconstruction, and analysis. XiaL and NL contributed to the CSFT system design, maintenance, and data capturing. FH performed the image preprocessing and format transformation. YC contributed to the NeuroGPS-Tree software maintenance. XW, WG, RC, and XiuL assisted in some experiments. TX, SZ, ZX, and HZ wrote the manuscript. AL participated in data processing and paper revision. All authors contributed to the article and approved the submitted version.

## Funding

We thank the Optical Bioimaging Core Facility of HUST for support with data acquisition. This work was financially supported by 973 programs (2015CB755603), the National Natural Science Foundation of China (Nos. 61721092, 81771376, and 91849121), and the Director Fund of WNLO.

## Conflict of Interest

The authors declare that the research was conducted in the absence of any commercial or financial relationships that could be construed as a potential conflict of interest.

## Publisher's Note

All claims expressed in this article are solely those of the authors and do not necessarily represent those of their affiliated organizations, or those of the publisher, the editors and the reviewers. Any product that may be evaluated in this article, or claim that may be made by its manufacturer, is not guaranteed or endorsed by the publisher.
