# Supplementary material for: Cross-Streams Through the Ventral Posteromedial Thalamic Nucleus to Convey Vibrissal Information
Source: Front Neuroanat. 2021 Oct 28;15:724861. doi: 10.3389/fnana.2021.724861 (PMC8582278; doi:10.3389/fnana.2021.724861)
Supplement: Supplementary file 4 [file Data_Sheet_1.pdf]

Supplementary Materials

This file includes:

Supplementary Figures S1 to S5 and their legends

Supplementary Tables S1 to S3

Supplementary Movies S1 to S3 and their legends

Other Supplementary Materials for this manuscript include the following:

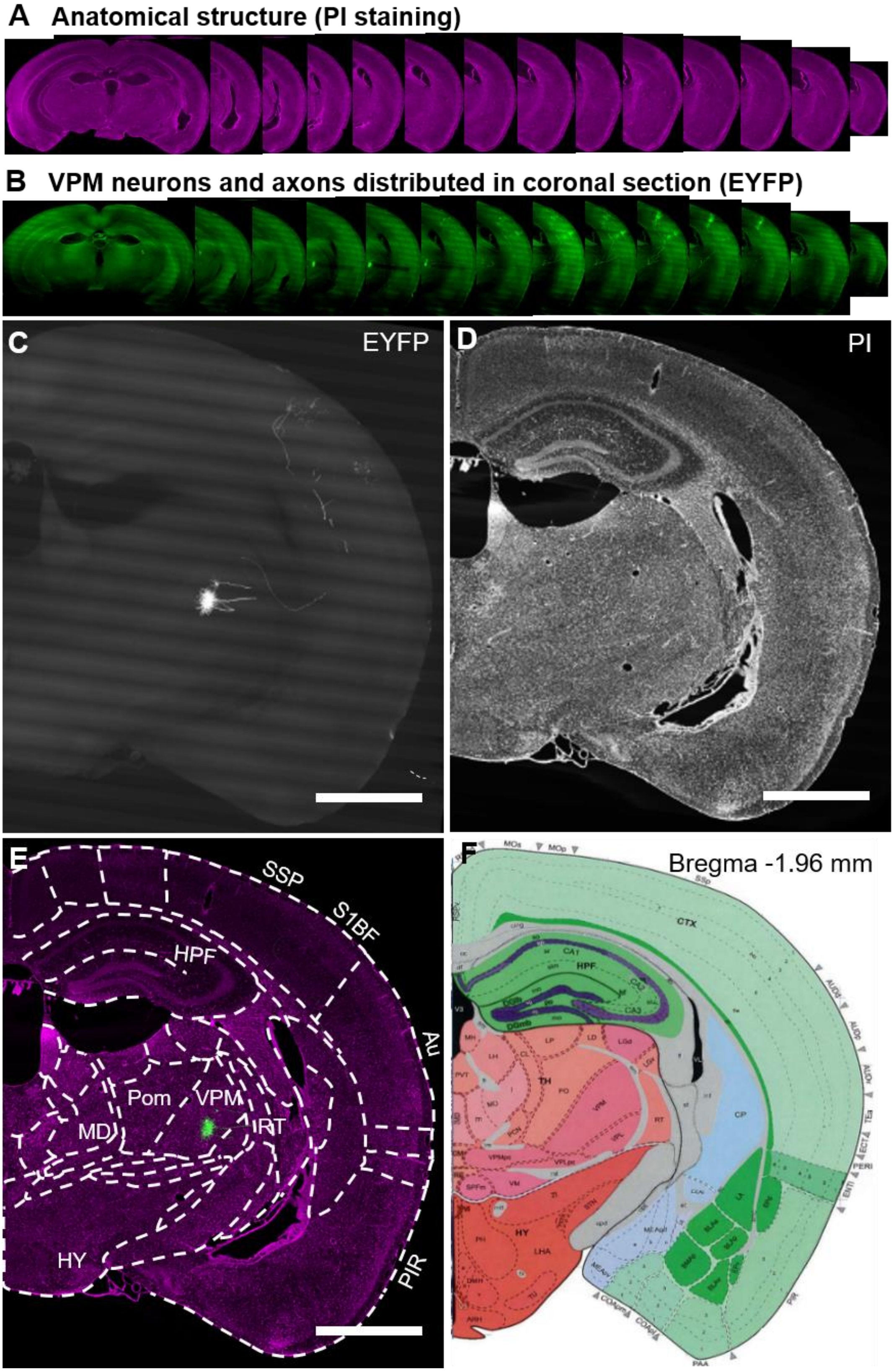

Figure S1. Fluorescence signals of labeled VPM neurons collected by CSFT system.

(**A**) A series of continuous PI channel sections for cytoarchitecture. (**B**) A series of continuous EYFP channel coronal sections for VPM neurons signals. Purple: 5 µm max-intensity projections. Green: 100 µm max-intensity projections. (**C-F**) Registration of raw images onto corresponding brain atlas. EYFP channel sections (**C**) and PI channel sections (**D**) were mixed to coronal section (**E**), which showing VPM nucleus and other brain regions according to corresponding sections of the Allen Reference Atlas (**F**). Scale bars: 1000 µm. Scale bars: 1000 µm (**C-F**).

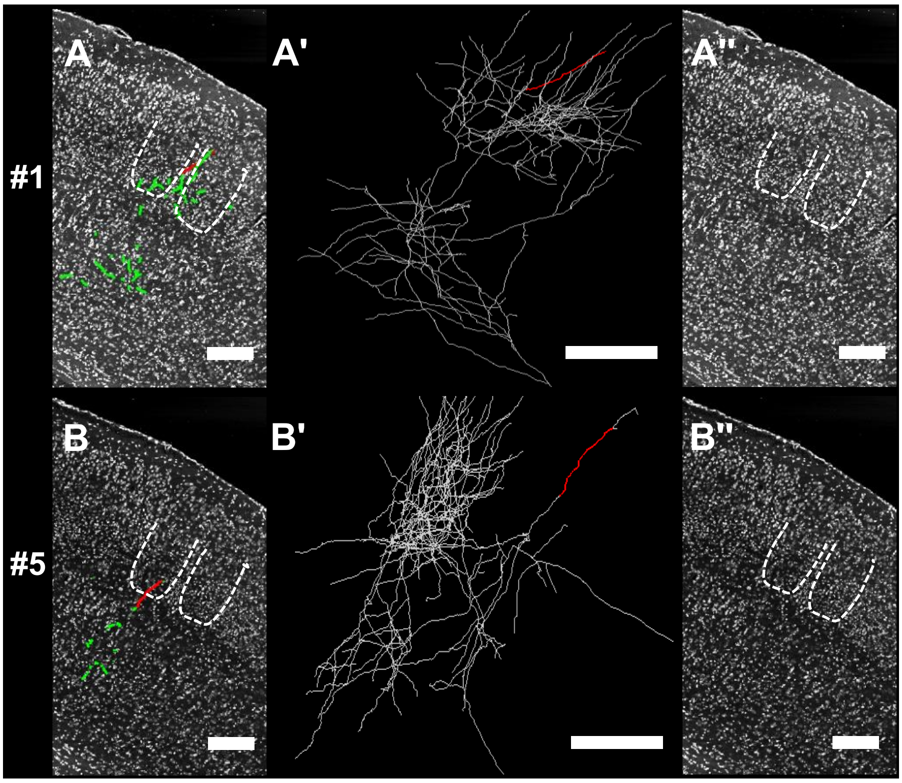

Figure S2. Innervation in multibarrels of individual VPMc neurons.

(**A-A'**) The branches of #1 VPMc neuron in two barrels and septal region. (**B-B'**) One branch of #5 in a barrel. The left pictures are coronal sections to reveal cytoarchitecture for region, the right pictures are the reconstructed VPMc neuronal morphology in left. White dash box: borders between barrels and septal areas. Red lines: some axons in barrel and septal regions. (**A-B**), scale bars: 500 µm, and (**A'-B'**), scale bars: 250 µm.

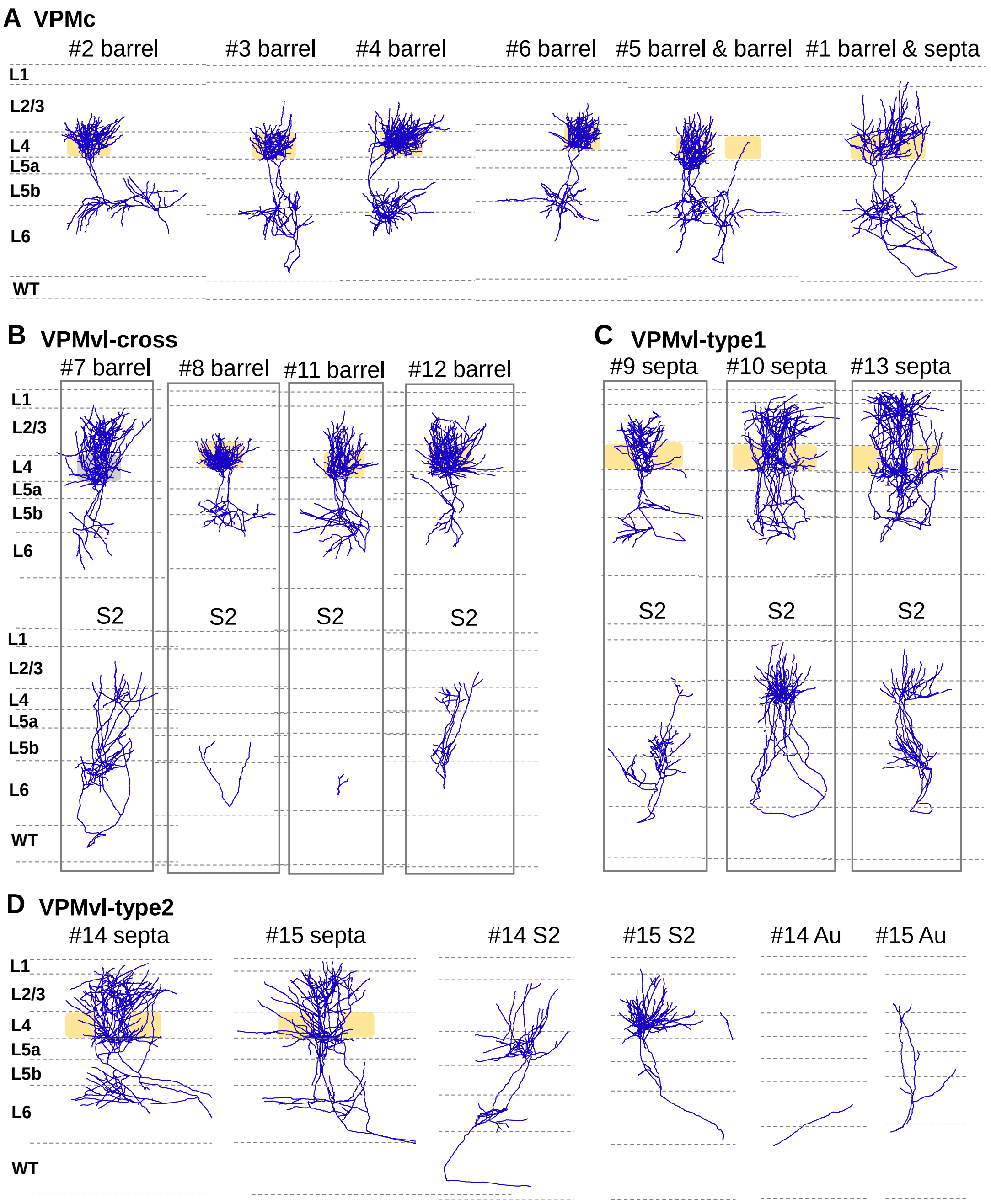

Figure S3. Morphology and patterns of thalamocortical projections of all 15 VPM neurons.

(**A**) Representative thalamocortical axons of individual VPMc morphologies in different layers. (**B**) Representative thalamocortical axons of individual VPMvl-cross morphologies in different layers of barrel columns and S2. (**C**) Representative thalamocortical axons of individual VPMvl-type1 morphologies in different layers of septal areas and S2. (**D**) Representative thalamocortical axons of individual VPMvl-type2 morphologies in different layers of septal areas, S2 and Au. Blue: cell axons. Grey box: Two clusters belong to the same cell. Grey dashed lines: Cortical layers. The

orientation of each reconstruction was re-adjusted according to the local cortical vertical axis (see more details in Methods).

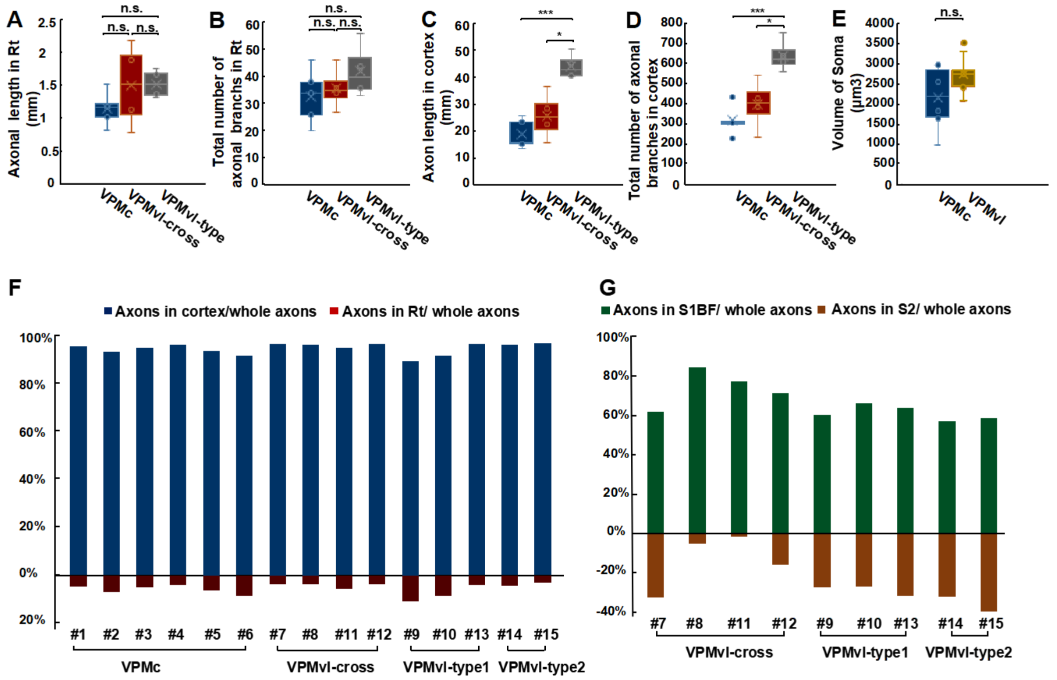

Figure S4. Characteristic analysis of three VPM subgroups in different areas.

Comparisons of axonal length in Rt (**A**) and total number of axonal branches in Rt (**B**) among VPMc, VPMvl-cross and VPMvl-type. Comparisons of axonal length in cortex (**C**) and total number of axonal branches in cortex (**D**) among VPMc, VPMvl-cross and VPMvl-type. (**E**) Comparisons of the soma volume between VPMc neurons and VPMvl neurons (including VPMvl-cross neurons, VPMvl-type1 neurons and VPMvl-type2 neurons). (**F**) Projection strengths in cortex and Rt nuclei for each individual neuron. (**G**) Projection strengths in S1BF and S2 for each individual neuron. * Represents p-value < 0.05, ** Represents p-value < 0.01, *** Represents p-value < 0.001, n.s. Represents no significant correlations.

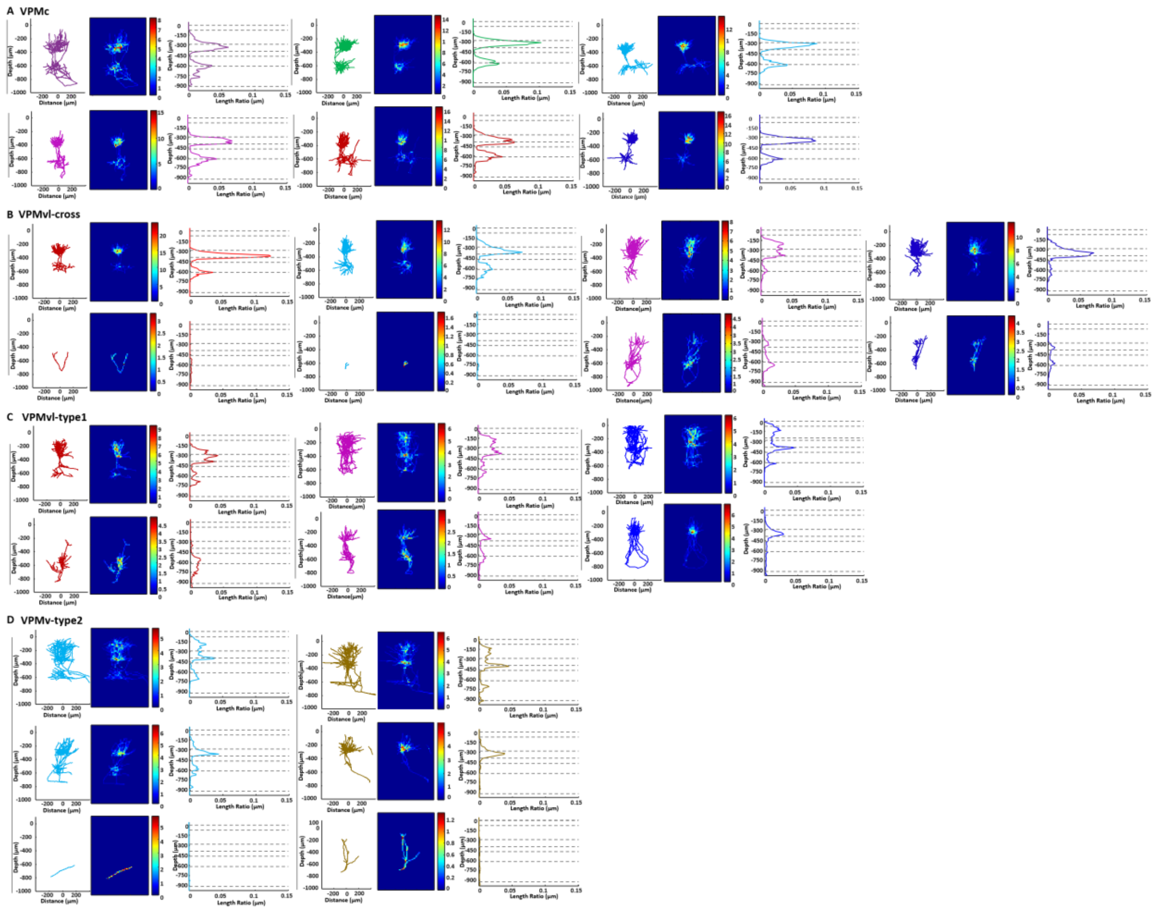

Figure S5. Quantitative analysis of all reconstructed VPM axons in different layers of cortex.

(A-**D**) Full list of different subgroup VPM neurons. The axon network (left) is converted into a 3D distribution (middle), and also shown as a 1D normalized profile along the vertical barrel column axis. A gray vertical line represents one VPM neuron and different colours represent distinct neurons in one subgroup. (**B**) The top row of VPM-cross neurons for axons is in barrel column, and the bottom for axons in S2 region, both belonging to the same neuron. (**C-D**) For VPMvl-type neurons, the top row is for typical axons in septa, and the middle for typical axons in S2 region, and also the bottom for the axons in Au region of the other two neurons. Those two categories have two similar profiles, and also similar to ones in S2 of VPMvl-cross neurons. Dashed horizontal lines represent cortical layer borders. Ratio presents density value. Scale bar: 200 μm.

Table S1. Data statistics of all 15 reconstructed VPM neurons.

Sample No.	Location	Category	Soma		Dendrite		full axons		Axons in Rt		Axons in barrel		Axons in septal		Axons in S2		Axons in Au	
			Surface	Volume (μm^3)	Length (μm)	Branches	Length (μm)	Branches	Length (μm)	Branches	Length (μm)	Branches	Length (μm)	Branches	Length (μm)	Branches	Length(μm)	Branches
1	VPMdm	VPMc	4069	1017	5917	140	24331	313	966	24	20919	278						
2			11993	2998	2619	78	20058	357	1193	46	13527	271						
3			10000	2582	2766	64	18592	269	826	34	13161	188						
4			12149	3007	3943	96	29523	463	1034	20	22977	383						
5			8016	1671	3357	64	27308	342	1554	26	20599	262						
6			7793	1840	2285	70	17972	340	1247	38	13640	297						
7	VPMvl	VPMvl-cross	12804	2825	3282	92	36497	547	1160	36	21564	375	11029	129				
8			9897	2438	3468	84	22345	389	801	27	17916	345	999	12				
11			10166	2484	4004	78	19978	277	1913	34	14700	229	242	5				
12			8385	2096	3284	82	28205	428	2227	46	19149	326	4078	50				
9		VPMvl-t1	11002	2750	3499	102	25796	402	1289	38			14826	238	6536	108		
10			11435	2859	6377	146	50561	755	1779	44			30678	524	14821	182		
13			13281	3320	5341	130	40371	610	1344	36			25449	394	10109	172		
14		VPMvl-t2	9785	2446	5944	130	48679	678	1393	33			27181	374	17955	268	2185	15
15			14210	3553	6398	170	4378	621	1699	56			23857	349	13064	198	866	1

Table S2. The VPM axon distribution from L4 to L1 in SIBF region.

The width and height from L4 to L1 in SIBF region					
**Location**	**Groups**	**No.**	**Length (μm)**	**Width (μm)**	**Height (μm)**
**VPMdm**	**VPMc**	1	531	392	325
		2	379	265.5	188
		3	275	180	252.9
		4	356	250	221
		5	598	190	225
		6	205	205	200
**VPMvl**	**VPMvl-cross**	7	425	313.6	355
		8	420	190	150
		11	494	264	277
		12	470	377	265
	**VPMvl-type1**	9	557	237	240
		10	612	272.5	310
		13	634	400	349
	**VPMvl-type2**	14	481	315	341.6
		15	494	264	277

Table S3. Abbreviations.

Abbreviations	
LD	Laterodorsal thalamic nucleus
LP	Lateral posterior thalamic nucleus
ml	medial lemniscus
Pom	Posterior thalamic nuclear group
Rt	Reticular thalamic nucleus
VPM	Ventral posteromedial thalamic nucleus
ZI	Zona incerta
VPL	Ventral posteralateral
LGv	Ventral part of the lateral geniculate complex
LGd	Dorsal part of the lateral geniculate complex
VMPpc	Ventral posteralateral nucleus of thalamus, parvicelllar part
Em	External medullary lamina of the thalamus
ml	medial lemniscus
pcn	paracentral nucleus
CL	Central lateral nucleus of the thalamus
LH	Lateral habenula
sm	stria medullaris
S1BF	Primary somatosensory area, barrel field
S2	Secondary somatosensory cortex
S1	Primary somatosensory cortex
Au	Primary auditory cortex
Sp5OVL	Spinal trigeminal nucleus, oral part, ventrolateral division
Sp5i	Spinal trigeminal nucleus, interpolar part
Pr5	Principal sensory trigemimal nucleus
L1, L2/3, L4, L5a, L5a, L6	cortical layers
PARN	Parvicellular reticular nucleus
IRN	Intermediate reticular nucleus
VII	Facial motor nucleus
icp	Inferior cerebellar peduncle
sptV	spinal tract of the trigeminal nerve
rust	rubrospinal tract
sctv	ventral spinocerebellar tract

Movie S1

Visualization of fiber distribution of VPM neurons in the whole mouse brain. This shows the 3D rotation of VPM neurons in the entire mouse brain. The resolution of the vertical plane is 2 μm × 2 μm.

Movie S2

The 3D reconstruction of all 15 VPM neurons innervating different regions in whole mouse brain. Reconstructed neurons were registered to a reference brain, overlain with Allen Common Coordinate Framework and show brainwide neuron projections. Four subgroups neurons are shown in different colors for different projection patterns. The raw data of the sections were imaged at 0.2 × 0.2 × 1.0 μm and then down-sampled to 4 × 4 × 20 μm to show the VPM structure.

Movie S3

The 3D reconstruction of soma localization of all 15 VPM neurons. Reconstructed neurons neurons are shown in different colors for soma localization. The raw data of the sections were imaged at 0.2 × 0.2 × 1.0 μm and then down-sampled to 4 × 4 × 20 μm to show the VPM were registered to a reference brain and overlain with Allen Common Coordinate Framework and show soma distribution of four subgroups in different VPM subregions. Four subgroups structure.
